# Beyond Tuberculosis: The Surprising Immunological Benefits of the Bacillus Calmette–Guérin (BCG) Vaccine in Infectious, Auto-Immune, and Inflammatory Diseases

**DOI:** 10.3390/pathogens14020196

**Published:** 2025-02-15

**Authors:** Magdalena Jurczak, Magdalena Druszczynska

**Affiliations:** 1 Department of Immunology and Infectious Biology, Faculty of Biology and Environmental Protection, Institute of Microbiology, Biotechnology and Immunology, University of Lodz, Banacha 12/16, 90-237 Lodz, Poland; magdalena.druszczynska@biol.uni.lodz.pl; 2 The Bio-Med-Chem Doctoral of the University of Lodz and Lodz Institutes of the Polish Academy of Sciences, University of Lodz, 90-237 Lodz, Poland; 3 Department of Microbiology and Experimental Immunology, MOLecoLAB: Lodz Centre of Molecular Studies on Civilisation Diseases, Medical University of Lodz, 92-215 Lodz, Poland

**Keywords:** Bacillus Calmette–Guérin, non-specific BCG effect, trained immunity

## Abstract

The Bacillus Calmette–Guérin (BCG) vaccine, best known for its role in preventing tuberculosis, has recently garnered attention for its broader immunomodulatory effects. By inducing trained immunity, BCG reprograms innate immune cells, enhancing their responses to various pathogens. This process, driven by epigenetic and metabolic reprogramming, suggests that BCG may have therapeutic potential far beyond tuberculosis. Emerging evidence points to its potential benefits in conditions such as autoimmune diseases, cancer, and viral infections. Furthermore, by modulating immune activity, BCG has been proposed to reduce chronic inflammation and promote immune tolerance. This review delves into the multifaceted role of BCG, highlighting its potential as a versatile therapeutic tool for managing a wide range of diseases.

## 1. Introduction

Recently, the protective, non-specific effects of the Bacillus Calmette–Guérin (BCG) vaccine have been recognised in the treatment of viral, autoimmune, and inflammatory diseases. The broad non-specific effects of BCG have attracted the attention of many scientists. As a result, research has begun to focus on developing new therapeutic approaches using the BCG vaccine. The mechanism responsible for the non-specific effects of the BCG vaccine is trained immunity, during which innate immune cells exhibit increased activity against pathogens other than *Mycobacterium* [1,2,3].

To explore the implications of this connection, we conducted a systematic review of studies published predominantly between 2020 and 2024, using keywords such as “trained immunity”, “BCG vaccine”, “non-specific BCG effect”, and “immunotherapy”. The studies were selected based on their critical importance in understanding the mechanisms of trained immunity, which play a significant role in the development of new therapeutic approaches.

The purpose of this review is to present current information on the BCG vaccine, with a focus on its underlying mechanisms, epidemiological evidence, and potential clinical applications. This review highlights recent reports on the potential use of the BCG vaccine in the treatment of viral infections, cancers, and autoimmune diseases. The goal of this study is to elucidate the broader health impacts of BCG vaccination, particularly in the context of emerging infectious diseases and evolving public health challenges.

## 2. The BCG Vaccine: Historical Context

The BCG vaccine was developed over a century ago by Dr. Albert Calmette and veterinarian Georges Guérin at the Pasteur Institute in Lille, France. After 13 years of cultivating *Mycobacterium bovis* (*M. bovis*) through 230 passages on a specialised potato–glycerol medium with 5% bile, the scientists obtained a strain that retained its antigenic properties while losing its original virulence [4]. In 1921, the BCG vaccine was administered for the first time to a human subject, a newborn whose mother had succumbed to tuberculosis. This pioneering procedure was soon followed by the widespread introduction of BCG vaccinations across Europe. After the Second World War, the World Health Organisation (WHO) expanded its vaccination recommendations globally [5]. Despite the long history of the tuberculosis vaccine, it remains the most widely used vaccine worldwide, with approximately 120 million newborns vaccinated annually. The cultivation of the original BCG strain on various microbiological media over time has led to numerous genetic alterations, resulting in the emergence of multiple genetically distinct sub-strains [6]. Genetic analyses, which have identified differences in deletions and insertions among the BCG strains, have classified these strains into four groups. Group 1, which shows the greatest similarity to the original BCG strain, includes BCG Russia, BCG Moreau, and BCG Japan. Group 2, which lacks the IS6110 gene located upstream of the phoP gene, includes BCG Sweden and BCG Birkhaug. Group 3 (BCG Glaxo, BCG Prague, and BCG Danish) emerged after 1931. The most recent group, designated as Group 4, includes the BCG TICE, BCG Frappier, BCG Phipps, BCG Connaught, and BCG Pasteur 1173 passaged strains [7].

The global incidence of tuberculosis has been significantly reduced due to the introduction of the BCG vaccination program. Moreover, scientific studies have begun to establish links between BCG vaccination and protection not only against tuberculosis but also against other pathogens. In 1927, Carl Näslund was the first to demonstrate that infants vaccinated with BCG had lower mortality rates compared to those who were not vaccinated [4]. A year later, Pearl (1928) discovered that tuberculosis patients had a lower incidence of cancer. Subsequent studies have shown that the BCG vaccine can also be effective in reducing child mortality [8,9,10].

In the following years, new findings emerged regarding the non-specific protective effects of BCG, which appeared to enhance host resistance to other infections. Following the implementation of the BCG vaccination program, a 40% reduction in mortality was observed in African countries, alongside a decline in the prevalence of respiratory diseases, malaria, sepsis, and leprosy [11,12,13,14]. The discovery of BCG’s broader protective effects prompted scientists to investigate the mechanisms underlying these non-specific reactions. In 2011, Netea et al. proposed the term “trained immunity” to describe this phenomenon [15]. Since then, research into the non-specific effects of BCG has gained renewed momentum.

## 3. Trained Immunity

The phenomenon in which the innate immune system exhibits immunological memory following repeated exposure to a pathogen is known as “immune training”. This concept is particularly intriguing, as it challenges the long-standing assumption that only the adaptive immune system could generate immunological memory, while innate immunity was traditionally considered incapable of forming a lasting imprint.

It is important to note, however, that the immune memories formed by these two systems are fundamentally different. Adaptive memory, mediated by long-lived T and B lymphocytes, arises from interactions between peptide-major histocompatibility complex (MHC) molecules and T and B cell receptors. This results in a highly specific and long-lasting response tailored to the encountered pathogen. In contrast, innate immune memory, or “trained immunity”, is rapidly induced following exposure to certain vaccines and antigens. These antigens are recognised by pattern recognition receptors (PRRs) on innate immune cells, triggering the reprogramming of myeloid cells, including macrophages, monocytes, natural killer (NK) cells, and dendritic cells (DCs) [16]. This reprogramming enables a heightened state of readiness upon subsequent encounters with pathogens, albeit in a less specific manner compared to adaptive memory.

The discovery of trained immunity not only broadens our understanding of immune system plasticity but also opens up potential therapeutic avenues. Harnessing this phenomenon could improve vaccine efficacy and offer novel strategies for combatting infections and even certain non-infectious diseases, such as cancer and autoimmune disorders.

### 3.1. Metabolic and Epigenetic Changes

**Figure 1:**
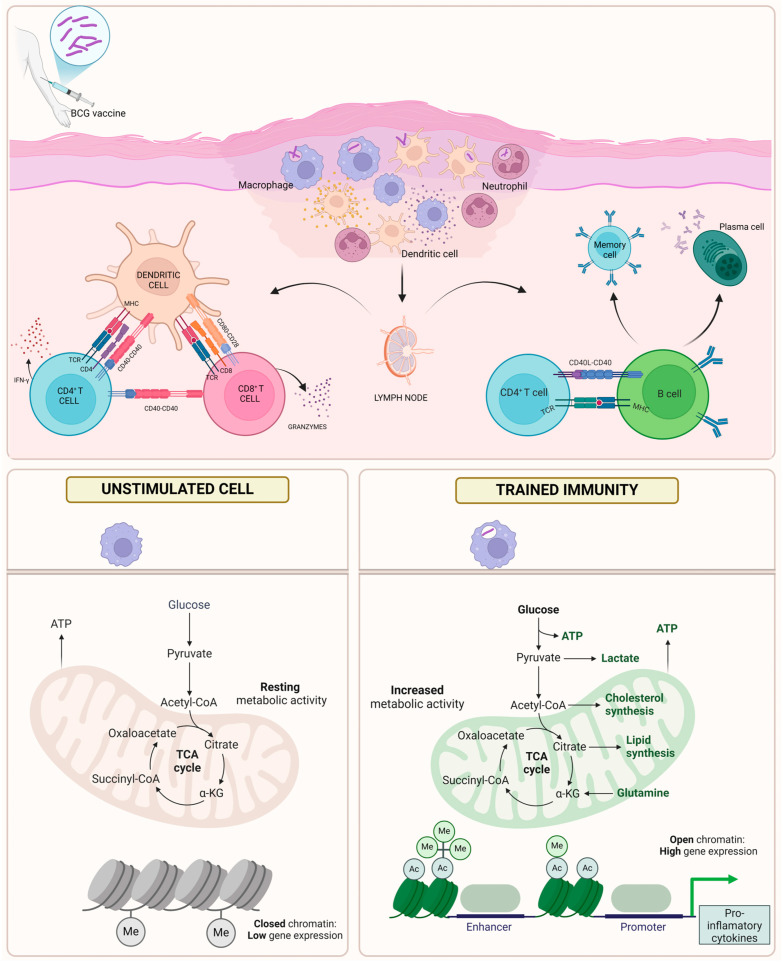
BCG-induced immunity training. The administration of the BCG vaccine causes the activation of macrophages, neutrophils, and dendritic cells. DCs then present antigens to CD4^+^ and CD8^+^ T lymphocytes, initiating an adaptive response, and CD4^+^ T lymphocytes stimulate B lymphocytes to produce memory cells and plasma cells. BCG vaccination induces epigenetic reprogramming in innate immune cells, such as monocytes and macrophages. Chromatin remodelling opens promoter regions, leading to high expression of pro-inflammatory cytokines. This response includes an increased production of cytokines like IL-1, TNF, and IL-6, which significantly strengthen the immune defence. Additionally, BCG induces metabolic reprogramming that elevates glycolysis, supplying the energy required for sustained cellular activation. Unlike adaptive immunity, BCG imparts a form of non-specific memory to the innate immune system, enabling it to mount faster and stronger responses to a variety of pathogens. Abbreviations: (Ac) acetylation, (Me) methylation, (ATP) adenosine triphosphate, (BCG) Bacille Calmette–Guérin, (TCA) tricarboxylic acid cycle, Acetyl-CoA (acetyl coenzyme A), (Succinyl-CoA) Succinyl-coenzyme A, interleukin (IL).

One of the most effective inducers of trained immunity is the BCG vaccine, which has the capacity to reprogram innate immune cells. This reprogramming is achieved through metabolic and epigenetic modifications that enhance the functionality of these cells during non-specific infections (Figure 1). The BCG vaccine induces trained immunity by binding to PRRs. In both in vivo and in vitro studies, Kleinnijenhuis et al. demonstrated that the BCG vaccine interacts with Nucleotide-binding Oligomerisation Domain 2 (NOD2), leading to an enhanced innate immune response. This interaction results in NOD2-dependent trimethylation of histone 3 at lysine 4 (H3K4me3) in the promoters of inflammatory cytokine genes [17]. The induction of trained immunity also involves the activation of the mevalonate pathway, which is further potentiated by the insulin-like growth factor I (IGF-I) pathway. Notably, the synthesis of both cholesterol and the metabolite mevalonate is essential for the induction of trained immunity [18]. These alterations are linked to post-translational modifications of histones within promoter regions and enhancer sequences. In response to training signals, histones undergo acetylation and methylation, leading to chromatin remodelling and gene expression changes that produce a stronger immune response. Furthermore, the stimulation of immune cells with BCG results in the induction of glycolysis and an increase in oxygen consumption, which are crucial for the full training of the immune system. When glucose metabolism is shifted from lactate production to the tricarboxylic acid (TCA) cycle, cytokine secretion is inhibited. The inhibition of glycolytic enzymes has been shown to prevent BCG-induced epigenetic alterations, such as H3K4me3 and histone 3 lysine 9 trimethylation (H3K9me3), indicating that enhanced glycolysis is a crucial component of trained immunity [19]. These metabolic and epigenetic modifications are regulated by the Akt/mTOR pathway [20]. Recent studies have further elucidated the role of metabolic pathways in trained immunity. For instance, research has shown that activating transcription factor 4 (ATF4) plays a significant role in regulating glucose metabolism in macrophages during sepsis. Knockdown of ATF4 impairs glycolysis and mediates immune tolerance by targeting hexokinase II (HK2) and hypoxia-inducible factor 1-alpha (HIF-1α) ubiquitination. This finding underscores the importance of glycolytic pathways in modulating immune responses [21].

Additionally, the concept of trained immunity has been explored in the context of other vaccines. For example, the diphtheria–tetanus–pertussis whole-cell (DTPw) vaccine has been shown to induce different programs of trained immunity in mice, suggesting that various vaccines may have distinct effects on the innate immune system [22].

These insights into the metabolic and epigenetic mechanisms underlying trained immunity highlight the complex interplay between cellular metabolism and immune function, offering potential avenues for therapeutic interventions in infectious and inflammatory diseases.

### 3.2. Cell Populations That Mediate Trained Immunity

The characteristics of trained immunity have been observed in numerous cells of innate immunity, including monocytes/macrophages, neutrophils, DCs, NK cells, innate lymphoid cells, haematopoietic stem cells (HSCs), microglia, and gamma delta (γδ) T cells [23,24,25,26,27,28]. Researchers have demonstrated that the immune-trained phenotype of monocyte-derived macrophages results in an elevated production of pro-inflammatory cytokines, including interleukin (IL)-6, IL-1β, and tumour necrosis factor (TNF) [29]. This regulation is associated with increased levels of H3K27 acetylation (H3K27ac) following BCG vaccination, specifically on the promoters of genes encoding cytokines involved in the inflammatory response [30]. Additionally, polymorphisms in the IL-1β gene have been observed to correlate with the intensity of the immune training response. Consequently, IL-1β has been identified as a key marker of BCG-induced innate memory in macrophages [30,31].

NK cells represent a vital element of the innate immune system, serving as a primary line of defence against pathogens. NK cells also possess the capacity to generate immune memory in response to a range of stimuli, including specific and non-specific inducers such as cytomegalovirus (CMV) infection, malaria, influenza vaccination, and BCG [26,32,33,34,35]. Studies have demonstrated that individuals who received the BCG vaccine and were subsequently stimulated with Mycobacterium tuberculosis, Staphylococcus aureus, or Candida albicans exhibited an elevated secretion of the pro-inflammatory cytokines TNF, IL-6, and IL-1β by NK cells. This phenomenon persisted for up to three months [17]. Furthermore, Suliman et al. observed that the re-administration of the BCG vaccine induced a prolonged NK cell response [36].

The induction of long-term innate immune memory is attributed HSCs, which exhibit greater longevity and intensified proliferation in the bone marrow compared to other innate immune cells. BCG vaccination has been shown to alter the transcriptional profile of HSCs, favouring myelopoiesis over lymphopoiesis. This modification results in the epigenetic reprogramming of macrophages [37]. Additionally, Cirovic et al. demonstrated that BCG vaccination in healthy individuals induces long-lasting transcriptomic alterations in hematopoietic stem and progenitor cells (HSPCs), further promoting myelopoiesis [38]. Recent studies have expanded on these findings. For instance, it has been observed that the modulation of HSC transcriptional profiles by BCG vaccination not only enhances myelopoiesis but also improves the resilience of macrophages against inflammatory and infectious challenges. Furthermore, the interplay between HSC reprogramming and metabolic pathways, such as glycolysis and oxidative phosphorylation, has been identified as a critical determinant of the trained immunity phenotype [39]. Novel insights into the role of chromatin remodelling complexes, such as the SWItch/sucrose non-fermentable (SWI/SNF) family, have elucidated their contribution to the stable epigenetic changes observed in innate immune cells post-BCG vaccination [40].

The capacity of the BCG vaccine to reprogram HSCs and subsequently enhance innate immune responses provides a promising foundation for the advancement of clinical vaccines and the therapeutic targeting of trained immunity. These findings open new avenues for the development of interventions aimed at improving immune responses in vulnerable populations and combatting a variety of infectious and non-infectious diseases. Further research is required to optimise vaccine strategies and fully elucidate the mechanisms underlying trained immunity, particularly in the context of varying genetic and environmental influences.

## 4. Non-Specific Effect

The BCG vaccine, originally developed for tuberculosis prevention, has demonstrated significant non-specific effects that extend beyond its primary purpose. Unlike many other vaccines, the BCG vaccine primarily activates cellular immunity, a mechanism that can also induce a phenomenon known as “trained immunity”. The components of the BCG vaccine, particularly its cell wall components, are recognised by PRRs, including Toll-like receptors (TLRs), NOD2, and C-type lectin receptors (CLRs). These interactions initiate a cascade of immune signalling pathways, leading to the production of pro-inflammatory cytokines, chemokines, and enhanced phagocytosis. This broad activation of the immune system is believed to contribute to the vaccine’s non-specific protective effects against various infections and even certain malignancies, including potential roles in modulating the immune environment in cancers such as lung cancer. Emerging research also highlights the potential of the BCG vaccine in immunotherapy, where its ability to stimulate innate and adaptive immune responses could complement checkpoint inhibitors or other targeted therapies. These findings underscore the versatility of the BCG vaccine and its broader implications for immune modulation and disease prevention [1].

### 4.1. Antiviral Effects

In recent years, an increasing number of novel respiratory pathogens have emerged, driving the occurrence of new pandemics and epidemics. As a result, the potential therapeutic application of immune training induced by the BCG vaccine is gaining attention as a strategy to combat emerging infections. It is hypothesised that the trained immunity elicited by the BCG vaccine could serve as an effective immunoprophylactic tool, enhancing the innate immune system’s response to viral infections. Current research is actively exploring the prophylactic effects of the BCG vaccine against various viral pathogens, including severe acute respiratory syndrome coronavirus 2 (SARS-CoV-2), respiratory syncytial virus (RSV), and influenza [41,42,43].

This line of investigation not only offers hope for mitigating the impact of respiratory viruses but also underscores the broader potential of trained immunity in public health. By harnessing the non-specific immune-boosting properties of vaccines like BCG, researchers aim to develop adjunct strategies that complement existing antiviral therapies and vaccination programs. However, while promising, the efficacy and safety of this approach require rigorous validation in diverse populations and across different viral contexts.

#### 4.1.1. SARS-CoV-2

The SARS-CoV-2 virus infects the respiratory tract, leading to acute disease. As of January 2025, it has caused approximately 704,753,890 cases worldwide, resulting in 7,010,681 deaths [44,45]. At the onset of the SARS-CoV-2 pandemic, scientists considered the BCG vaccine, known for inducing non-specific host responses to certain viral infections, as a potential prophylactic against SARS-CoV-2. This hypothesis was initially supported by observations of lower infection rates in countries with mandatory tuberculosis vaccination policies. Several epidemiological studies provided evidence supporting this idea. However, subsequent experimental studies questioned the protective effect of the BCG vaccine against SARS-CoV-2 infection [46].

In animal models, Zhang et al. demonstrated that intravenous BCG vaccination did not prevent infection but reduced SARS-CoV-2 replication and the pro-inflammatory response by activating the glycolysis pathway and promoting myeloid cell polarisation [47]. Similarly, Singh et al. found that BCG vaccination had a beneficial impact on SARS-CoV-2 infection in animals, inducing reprogramming of myeloid cells and lymphocytes, thereby preventing virus-induced lymphopenia [41]. Hilligan et al. reported that intravenous BCG vaccination reduced pulmonary pathology, including granuloma formation in alveolar septa [48]. In contrast, Kaufmann et al., 2022, observed no significant protective effect of BCG vaccination against SARS-CoV-2, suggesting that differences in BCG strains and administration routes might explain these discrepancies [43]. The route of BCG vaccine administration appears to influence its efficacy. In mice, subcutaneous administration did not affect the course of SARS-CoV-2 infection, whereas intravenous administration increased the likelihood of a protective effect by activating CD4^+^, CD8^+^, and NKT cells [48]. Retrospective and ecological studies on the association between BCG vaccination and SARS-CoV-2 incidence have yielded inconclusive results. For instance, a study in the United Arab Emirates found that healthcare workers who received a second BCG dose did not contract SARS-CoV-2, while non-vaccinated individuals did [49]. Conversely, the BCG-CORONA study in South Africa indicated that BCG revaccination was associated with an elevated risk of severe SARS-CoV-2 infections compared to a placebo [50]. A Brazilian study found that the revaccination of healthcare workers with the BCG-Moscow vaccine did not provide protection against SARS-CoV-2 infection [51].

Some researchers have explored the potential of BCG vaccination as an adjuvant to SARS-CoV-2 vaccines. It has been observed that administering the BCG vaccine prior to anti-SARS-CoV-2 vaccination results in a superior immune response, characterised by elevated antibody titres and increased levels of pro-inflammatory cytokines, including TNF and IL-1β [52,53]. The inconsistency in study results regarding the protective effect of BCG vaccination can be attributed to variables such as gender distribution, age, participant numbers, BCG strains, administration methods, dosage, and timing relative to pathogen exposure. Additionally, the protective effect of BCG may vary depending on the specific pathogen. Kaufmann et al., 2022, noted that while BCG vaccination did not protect against SARS-CoV-2, it reduced morbidity and mortality associated with influenza A virus [43]. Notably, aerosol administration of the BCG-Danish vaccine to rhesus macaques resulted in an increased production of IL-6, IL-1β, and TNF as well as elevated numbers of circulating monocytes and activated γδ T cell populations, suggesting that BCG aerosol vaccination may induce innate immune mechanisms and prepare unconventional T-cell populations [54].

Recent investigations have highlighted the potential role of the BCG vaccine in mitigating the effects of COVID-19. A study by Jalalizadeh et al., 2025, explored the impact of BCG vaccination administered during the active phase of COVID-19. The findings suggest that the vaccine may help prevent the development of long COVID by enhancing trained immunity mechanisms that reduce systemic inflammation and promote faster immune recovery. This study emphasises the potential of BCG vaccination as a therapeutic intervention during COVID-19 infection to address lingering symptoms and complications associated with long COVID [55]. In addition, the preliminary findings of the randomised controlled trial Bacillus Calmette–Guérin Vaccination as Defense Against SARS-CoV-2 (BADAS), which aimed to assess the efficacy of BCG vaccination in preventing SARS-CoV-2 infection and reducing disease severity among high-risk individuals, highlighted the importance of BCG-induced immune training as a cost-effective and broadly applicable strategy for pandemic preparedness [56]. Both studies contribute valuable insights into the broader applications of the BCG vaccine in managing not only SARS-CoV-2 infection but also its long-term effects, emphasising the need for further research to confirm these findings and optimise intervention strategies.

#### 4.1.2. RSV

RSV is a leading causative agent of lower respiratory tract infections and is responsible for a substantial burden of hospitalisations among newborns and young children worldwide. The clinical spectrum of RSV infection ranges from mild to severe, with manifestations including alveolitis, bronchiolitis, and pneumonia [57]. Globally, RSV is estimated to cause approximately 33.1 million cases of acute respiratory infections in children under five years of age annually, with more than 3.6 million cases requiring hospitalisation and over 100,000 fatalities [58].

Epidemiological studies have revealed that BCG vaccination of newborns provides protection not only against tuberculosis but also against other respiratory infections, including RSV [42]. Research has demonstrated that BCG vaccines, particularly when engineered to express RSV antigens, show promising outcomes in conferring immunity against RSV. Recombinant BCG strains expressing RSV antigens (rBCG-N-hRSV) have been shown to promote protective Th1-type immunity in mice, characterised by a robust activation of RSV-specific T cells that produce IFN-γ and IL-2 [59]. Studies by Cautivo et al. and Céspedes et al. further highlighted that rBCG immunisation enhances the early recruitment of CD4^+^ and CD8^+^ T cells to the lungs alongside the secretion of IFN-γ and IL-17, which are critical in controlling RSV infection [57,60]. Espinoza et al. observed that immunisation with rBCG-N-hRSV also confers neuroprotective effects by preventing RSV-related central nervous system complications in murine models [61]. Additionally, Wang et al. demonstrated that administering the BCG vaccine at birth, followed by a formalin-inactivated RSV vaccine, reduces the severity of RSV-related respiratory disease and enhances long-term protection through trained immunity and balanced Th immune memory [62]. Importantly, rBCG-N-hRSV has been found to induce not only cellular but also humoral immunity, with the production of antibodies targeting RSV proteins [63]. This dual immune response is particularly significant, as rBCG-N-hRSV is currently the only RSV vaccine designed specifically for newborns—a demographic that represents the majority of RSV-related hospitalisations, especially infants under six months of age [63]. These findings highlight the importance of integrating novel recombinant vaccines into immunisation programs to mitigate the global burden of RSV, especially in vulnerable populations.

#### 4.1.3. Influenza Virus

Influenza is a major global health concern, with approximately one billion cases reported worldwide each year [64]. Of these cases, an estimated 3 to 5 million people develop severe influenza, resulting in a mortality rate of 290,000 to 650,000 deaths annually. Influenza A virus, the causative agent of acute respiratory infections, is highly contagious, spreading through direct or indirect contact. Clinical manifestations of infection typically include cough, fever, sore throat, body aches, and fatigue [64].

Recent research has explored the protective effects of the BCG vaccine in response to viral infections, including the influenza A virus. Kaufmann et al., 2022, demonstrated that the BCG vaccine provides significant protection against influenza A virus infection in animal models. Specifically, Syrian hamsters vaccinated with BCG showed a marked reduction in viral titres in the lungs following infection with the influenza A virus [43]. Further advancements have focused on developing vaccines utilising the BCG cell wall skeleton (BCG-CWS) as an adjuvant. Studies have revealed that BCG-CWS induces robust humoral and cellular immune responses, characterised by elevated levels of immunoglobulin G (IgG)1 and IgG2a antibodies. Additionally, BCG-CWS has been shown to regulate pathological inflammation and suppress excessive Th1 cell proliferation during influenza virus exposure [65].

Building upon these findings, recent studies corroborate the protective effects of the BCG vaccine. Tran et al. highlighted that intravenous administration of the BCG vaccine conferred substantial protection against subsequent influenza A virus infection in mice [66]. A critical mechanism underlying this protection involves the enrichment of conventional αβ CD4^+^ effector memory T cells in circulation and lung parenchyma. These cells express high levels of the CX3C chemokine receptor 1 (CX3CR1^hi^), playing a pivotal role in mitigating the early stages of viral infection independently of an antigen. The enhanced efficacy of these CX3CR1^hi^ T cells is attributed to their intensive production of interferon-γ, which activates alveolar macrophages and promotes sustained antimicrobial activity. This interplay between innate and adaptive immune responses underscores the broad-spectrum protection afforded by the BCG vaccine [67].

These findings collectively highlight the potential of the BCG vaccine and its derivatives as effective tools not only for preventing tuberculosis but also for combatting viral infections such as influenza A. Future research should prioritise clinical trials to validate these effects in humans and explore the utility of BCG-based strategies in pandemic preparedness and respiratory virus management.

### 4.2. Immunotherapy for Cancer

Cancer remains the leading cause of mortality in humans, with an estimated 20 million new cases and 9.7 million deaths reported in 2022 [68]. The anti-cancer effects of BCG were first investigated as early as 1929, when researchers observed a reduced incidence of cancer in autopsy cases of individuals with tuberculosis [69]. Initially, it was hypothesised that the BCG vaccine induced an intracellular inflammatory response, resulting in tumour shrinkage. The utilisation of immunotherapy in cancer treatment has emerged as a promising strategy for inhibiting tumour growth and metastasis by harnessing the immune system’s response [70]. However, traditional cancer therapies often rely heavily on T-cell activation, which may prove insufficient in certain cases due to the suppressive nature of the tumour microenvironment, which inhibits robust immune responses [71]. This limitation highlights the potential of “trained immunity”, a phenomenon where the innate immune system develops a form of immunological memory [72]. BCG vaccination, whether used as monotherapy or in combination with other treatments, can reprogram the tumour microenvironment, transforming it into a setting more conducive to effective anti-tumour activity. This approach fosters pro-inflammatory conditions, which support the mobilisation of immune responses and sustain the activity of tumour-targeting T cells [73].

By leveraging both the direct and indirect effects of BCG vaccination, cancer immunotherapy may overcome the barriers posed by the tumour microenvironment, offering a novel pathway for improved clinical outcomes in cancer management.

#### 4.2.1. Trained Immunity in Cancer Immunotherapy

The training of the immune system is influenced by various factors, notably the BCG vaccine, which has been shown to enhance the innate immune response by strengthening NK cells and activating T lymphocytes. Trained innate immune cells, including macrophages, monocytes, and NK cells, exhibit heightened reactivity and an increased capacity to respond swiftly to stimuli, such as signals from cancer cells. This enhanced responsiveness may improve the efficacy of therapies like chimeric antigen receptor (CAR)-T cell therapy, which aims to activate T lymphocytes or NK cells against tumour cells [74]. Epigenetic modifications, particularly histone alterations, are closely associated with immune training. Specific pharmacological agents can influence histone methylation, thereby augmenting anti-tumour responses. For instance, lysine demethylase 6B (KDM6B), an enzyme responsible for histone demethylation, can support a pro-inflammatory response, potentially enhancing the effectiveness of anti-cancer therapies [75]. Moreover, the phenomenon of trained immunity depends on a fully functional autophagy mechanism. The inhibition of autophagy has been observed to impede epigenetic modifications, thereby limiting the development of trained immunity. This is exemplified by the reduced responsiveness of patients with mutations in autophagy-related genes, such as autophagy-related 2B (ATG2B), to BCG immunotherapy for bladder cancer [76]. Understanding these intricate mechanisms offers promising avenues for enhancing cancer immunotherapy by modulating trained immunity, epigenetic landscapes, and autophagic pathways.

#### 4.2.2. Bladder Cancer

Bladder cancer represents the 9th most common cancer worldwide, with a higher prevalence in males than females. The majority of patients are diagnosed with non-muscle invasive bladder cancer (NMIBC); however, approximately 20% present with muscle-invasive bladder cancer (MIBC). Treatment typically includes endoscopic tumour removal, intravesical adjuvant therapy, radiotherapy, and chemotherapy [77].

In 1929, Pearl’s observations led to the development of the BCG vaccine, which has demonstrated efficacy against bladder cancer [78]. BCG immunotherapy is now considered the gold standard for the adjunctive treatment of NMIBC. This therapy involves the intravesical administration of a suspension of live attenuated BCG bacteria at a specific concentration, with dosage and duration tailored to the patient’s condition [79]. Research indicates that BCG immunotherapy not only stimulates the immune system to suppress neoplastic cells but also offers superior protection against disease recurrence [80]. Despite extensive studies over several decades, the precise mechanisms remain unclear. It is hypothesised that intravesical BCG administration triggers both local and systemic immune responses, activating urothelial cells, macrophages, dendritic cells, lymphocytes, and neutrophils [70]. Furthermore, it was noted that combination treatment against PD-1 in patients with NMIBC bladder cancer could potentially increase the number of patients responding to treatment with the BCG vaccine [81]. In contrast, Patwardhan et al., 2024, showed that glutathione-S-transferase Theta 2 (Gstt2) expression was associated with a better response to BCG intravesical immunotherapy in patients with NMIBC [82]. In vitro studies have shown that BCG can increase the production of cytokines such as IL-6, IL-8, and granulocyte–macrophage colony-stimulating factor (GM-CSF) o [83,84,85]. In vivo studies in humans have observed that BCG administration induces the production of neutrophils, macrophages, monocytes, NK cells, and T and B lymphocytes, accompanied by elevated levels of IL-1β, IL-8, IL-15, IL-18, and CXC chemokine ligand (CXCL)10, CCL2, and CCL3. The rationale for using cytokines in the treatment of bladder cancer with BCG is known to be based on their ability to induce a Th1-type immune response, crucial for the elimination of cancer cells. Cytokines such as IL-2, IL-12, IL-18, TNF, GMCSF, and IFN-α work together to stimulate the production of IFN-γ to enhance the immune response. The synergistic effect of cytokines supports the development of an immune response and improves the efficacy of treatment [86,87,88].

Despite its effectiveness, BCG therapy is associated with certain limitations, including significant variability in patient response and the risk of recurrence. Addressing these challenges requires further research to unravel the complex interplay between host immunity, tumour biology, and the mechanisms of BCG action. Such insights could pave the way for more personalised and effective therapeutic strategies in bladder cancer management.

#### 4.2.3. Melanoma

Malignant melanoma, the most aggressive form of skin cancer, is being diagnosed in an increasing number of individuals annually. In 2024, there were 100,640 cases reported globally, including 8290 deaths [89]. An early diagnosis of melanoma significantly improves a patient’s prognosis. However, approximately 10% of cases are diagnosed at an advanced stage, characterised by metastases and inoperability [90].

The first attempts to use the BCG vaccine in the treatment of melanoma date back to the 20th century, when Morton et al. observed tumour regressions in cancer patients treated with BCG [91]. Several therapeutic approaches utilising the BCG strain have since been explored for melanoma treatment. These include the administration of the BCG vaccine alone, in combination with autologous tumour cells, or via direct injection into tumour lesions [92]. However, combining the BCG vaccine with cytotoxic drugs, cancer vaccines, or cytokine therapies has not demonstrated significant benefits for melanoma patients [93,94]. Conversely, Faries et al. reported that co-administration of BCG with the anti-tumour vaccine Canvaxin elicited a robust anti-tumour response and prolonged patient survival [95]. Furthermore, promising results have been observed in melanoma patients treated with BCG combined with TLR agonists [96]. Nishida et al. focused on the BCG-CWS, which acts as a potent immune adjuvant due to its abundance of TLR2 ligands. Their study demonstrated an increased production of monocytes and neutrophils in response to BCG-CWS. Notably, some patients exhibited CD4^+^ T cell activation and differentiation into memory phenotypes, enhancing their anti-tumour response [97].

The use of BCG as an immunomodulator in melanoma therapy leads to the production of inflammatory cytokines (IFN-γ, TNF, TNF-β, IL-15, IL-1β, IL-6, and IL-32) and chemokines (CCL2, CCL18, CXCL9, CXCL10, and CXCL11). Additionally, stress-induced molecules such as Butyrophilin Subfamily 3 Member A1 (BTN3A1) and MHC class I-related chain B (MICB) are induced, resulting in melanoma metastasis regression [98]. BCG-induced anti-tumour responses, while effective, remain poorly understood at the immune mechanism level. Borges et al. investigated the immune profile of B16-F10 mouse melanoma cells and highlighted the pivotal role of MyD88 signalling in cytokine production during BCG immunotherapy. Their findings revealed that MyD88 promotes the recruitment and activation of various immune cells, including M1-like tumour-associated macrophages, neutrophils, dendritic cells, CD4^+^ and CD8^+^ T cells, NK cells, and NKT cells, within the tumour microenvironment [99].

Recent studies have explored the potential of combining BCG with immune checkpoint inhibitors, such as anti-PD-1/PD-L1 antibodies, to enhance the therapeutic efficacy in melanoma patients. This combinatorial strategy aims to overcome resistance mechanisms associated with advanced melanoma and has shown promising preclinical results [94]. Additionally, there is growing interest in the use of BCG-derived exosomes as carriers for tumour-specific antigens. These exosomes can potentiate the immune response against melanoma, providing a novel strategy for delivering targeted immunotherapy [94].

Emerging research continues to uncover novel insights into the immunological mechanisms underlying BCG-mediated anti-tumour responses, opening new avenues for its integration into advanced melanoma immunotherapies.

#### 4.2.4. Lung Cancer

Lung cancer is the second most common cancer worldwide, diagnosed in 2,480,675 individuals in 2022 [100]. Unfortunately, it has a low survival rate and accounts for the highest number of cancer-related deaths due to its poor prognosis and challenges in early detection [100]. In 1976, McKneally et al. conducted the first study investigating the use of BCG in lung cancer treatment. They demonstrated that a single postoperative intrapleural dose of BCG was well tolerated at restricted doses in patients following lung resection and improved outcomes in patients with stage I lung cancer [101]. Subsequent studies focused on the BCG cell wall, showing that BCG-CWS alleviated lymphocyte suppression in lung cancer patients and prolonged survival in stage III and IV cases [65]. However, conflicting reports have emerged about the positive effects of the BCG vaccine, with some suggesting that BCG might stimulate tumour growth in lung cancer [102,103,104]. On the other hand, Moreo et al. demonstrated in mouse models that intravenously administered BCG induced CD8^+^ T cell and NK cell responses against lung tumour cells [105]. Furthermore, the combination of BCG therapy with an immune checkpoint blockade (ICB) showed beneficial effects. The enhancement of systemic tumour cell-specific cytotoxic responses was shown [105]. Supporting this, Lapieza et al. provided strong evidence that the intravenous administration of BCG in mice activates robust NK and CD8^+^ T cell responses, which significantly enhances anti-tumour immunity in the lungs and effectively overcomes resistance to existing immunotherapies. Their findings suggest a promising role for BCG in combination strategies to enhance the efficacy of checkpoint inhibitors and other immune-targeted therapies [106].

Other studies have investigated the use of mitumomab (BEC-2), a murine monoclonal antibody targeting ganglioside GD3, in combination with the BCG vaccine for treating small-cell lung cancer. Unfortunately, phase III clinical trials showed no significant benefit for patients receiving mitumomab and BCG [107,108].

### 4.3. Immunotherapy for Autoimmune Diseases

The prevalence of autoimmune diseases is steadily increasing, currently affecting approximately 3–8% of the global population [109]. This rise is attributed to a variety of environmental factors, including pollution, changes in dietary habits, and possibly the increasing prevalence of sedentary lifestyles and stress. These factors contribute to a heightened immune dysregulation, triggering autoimmune responses in susceptible individuals. Since research has shown that the BCG vaccine enhances glycolysis in trained human monocytes—a metabolic reprogramming process that strengthens immune memory and functionality—it has sparked significant interest in its potential application as a therapeutic tool for autoimmune diseases [110]. By reprogramming innate immunity, fostering an anti-inflammatory environment, and potentially restoring immune balance, the BCG vaccine represents a novel and promising approach to addressing the dysregulated immune response characteristics of autoimmune disorders.

#### 4.3.1. Multiple Sclerosis (MS)

An estimated 2.9 million people worldwide are affected by multiple sclerosis (MS), a chronic demyelinating and inflammatory disease of the central nervous system (CNS) characterised by damage to nervous tissue. This condition disrupts communication between the brain and other parts of the body, leading to a wide range of neurological symptoms and progressive disability [111].

Studies on animal models of MS, such as experimental autoimmune encephalomyelitis (EAE), have demonstrated that the administration of a killed Mycobacterium strain can inhibit demyelination and reduce disease severity. These promising preclinical findings have sparked interest in the potential therapeutic effects of the Bacillus Calmette–Guérin (BCG) vaccine for MS [112]. Clinical investigations have provided encouraging results. A pivotal study by Ristori et al. demonstrated that the intradermal administration of the BCG vaccine to patients with relapsing–remitting MS (RRMS) reduced the number of active lesions visible on magnetic resonance imaging (MRI) without causing adverse effects [113]. Follow-up studies by the same group revealed that the early administration of BCG in the disease course could delay MS progression. Additionally, BCG vaccination appeared to block tissue damage, promote neuroinflammation repair, and reduce the risk of new MRI lesions in patients with clinically isolated syndrome (CIS), a condition that often precedes MS [114,115]. The exact mechanisms through which BCG exerts its protective effects in MS remain unclear. However, recent research has highlighted the vaccine’s ability to induce metabolic and immunological changes beneficial in MS. BCG vaccination has been shown to upregulate glycolysis and modulate cellular respiration pathways, processes often impaired in patients with relapsing–remitting multiple sclerosis (RRMS). This metabolic reprogramming enhances immune cell functionality, potentially fostering a better regulation of neuroinflammatory responses [116]. Furthermore, BCG’s role in “trained immunity”, which involves epigenetic reprogramming of innate immune cells, may significantly impact its therapeutic potential. This mechanism has been shown to promote balanced immune responses and reduce autoreactive activity driving CNS inflammation in MS. Notably, monocytes and macrophages subjected to BCG-induced trained immunity exhibit enhanced glycolysis, increased cytokine production, and improved immune tolerance—all factors potentially contributing to CNS repair [117]. Recent studies have suggested potential interactions between trained immunity induced by BCG and cytokines from the IL-1 family, which play a central role in modulating inflammation in autoimmune diseases. The broader benefits of BCG-induced metabolic switching, such as promoting aerobic glycolysis, have been discussed in autoimmune and neurodegenerative diseases. This metabolic adaptation, transitioning from oxidative phosphorylation to glycolysis, may underlie the improved immune regulation and neuroprotection observed in MS patients [118]. These findings collectively emphasise the potential of BCG as a safe and cost-effective therapeutic option for MS, particularly in the early stages of the disease. However, larger randomised controlled trials are essential to confirm these benefits, unravel the underlying mechanisms, and optimise treatment protocols.

#### 4.3.2. Type 1 Diabetes (T1D)

Type 1 diabetes (T1D) is a chronic autoimmune disease affecting approximately 9.4 million individuals worldwide, with projections indicating that this number will rise to 17.4 million by 2040. This condition is characterised by the targeted destruction of pancreatic β-cells by autoreactive CD8^+^ T cells, leading to insulin deficiency and hyperglycaemia. Despite extensive research, the precise aetiology of T1D remains elusive. Current evidence suggests that the disease arises from a complex interplay of genetic predisposition and environmental triggers, including infections, diet, and microbiome composition [119]. Interestingly, epidemiological observations have revealed a lower incidence of T1D among individuals vaccinated with BCG. These findings spurred preclinical studies using the non-obese diabetic (NOD) mouse model. In these studies, the administration of complete Freund’s adjuvant (which contains heat-killed BCG) or the BCG vaccine alone significantly reduced the development of diabetes [120]. These results laid the foundation for subsequent investigations into the immunomodulatory effects of BCG in T1D.

One of the proposed mechanisms involves the induction of TNF, which selectively eliminates insulin-autoreactive T cells while sparing healthy T cells [121,122,123]. Additionally, TNF exposure has been shown to enhance the proliferation of regulatory T cells (Tregs), which suppress autoreactive immune responses. Beyond immune modulation, BCG-induced TNF has been implicated in pancreatic regeneration, mediated by increased levels of C-peptide, a marker of endogenous insulin production and β-cell function [115,124]. Further research has highlighted the role of BCG vaccination in altering metabolic pathways within the immune system. Patients with T1D who received BCG vaccinations demonstrated significant reductions in haemoglobin A1c levels, bringing them closer to normal ranges. Mechanistically, this improvement was linked to a shift in immune cell metabolism from oxidative phosphorylation (OxPhos) to aerobic glycolysis, a phenomenon often referred to as the “Warburg effect.” This metabolic reprogramming may enhance immune tolerance and β-cell resilience [118,125]. More recent studies have underscored the potential of BCG as a therapeutic agent in T1D, not only for its immune-regulatory effects but also for its ability to stabilise blood glucose levels and improve overall metabolic health. The vaccine’s broad effects on innate immune training, particularly through epigenetic and transcriptional reprogramming, may also contribute to its therapeutic benefits. This expanding body of evidence positions BCG as a potential adjunct therapy for T1D, warranting further clinical trials to optimise dosing regimens and evaluate long-term safety and efficacy [124,125,126] (Table 1).

## 5. Conclusions

The trained immunity induced by the BCG vaccine represents a promising avenue for advancing our understanding of the innate immune response and its potential for therapeutic applications. Originally developed to prevent tuberculosis, the BCG vaccine has been observed to elicit a broad spectrum of non-specific protective effects. These effects are attributed to epigenetic and metabolic reprogramming in innate immune cells, such as monocytes and macrophages, which enhance their ability to respond to subsequent infections. Remarkably, these effects can persist for months or even years, positioning BCG as a unique and efficacious immunomodulatory agent. Studies have demonstrated that BCG vaccination is associated with a reduction in the incidence and severity of viral infections, including respiratory illnesses. This is likely mediated through the activation and enhanced functional capacity of macrophages and monocytes, enabling them to effectively neutralise viral antigens. Moreover, BCG has shown promise as an immunotherapy for certain cancers, particularly bladder cancer. The vaccine’s ability to modulate the tumour microenvironment facilitates the recruitment and activation of immune cells that directly target and destroy cancer cells. In addition to its direct cytotoxic effects on pathogens and tumour cells, trained immunity induced by BCG plays a critical role in fine-tuning the inflammatory response. By regulating the activation of innate immune cells, BCG can mitigate excessive inflammation, which is a hallmark of many chronic and infectious diseases. This dual capacity to enhance host defence while limiting hyperinflammatory responses underscores the therapeutic versatility of the vaccine.

Despite significant advancements in understanding the mechanisms underlying BCG-induced trained immunity, several questions remain. Key areas for future research include the durability of these immunological effects, the potential variability in response among individuals, and the long-term safety of repeated or off-label BCG administration. Furthermore, exploring the interplay between trained immunity and adaptive immune responses could open new frontiers in vaccine development and immunotherapy. BCG’s non-specific benefits highlight its broader significance beyond tuberculosis prevention, offering insights that may inform strategies for combatting emerging infectious diseases, inflammatory conditions, and cancer. As our understanding of trained immunity deepens, BCG serves as a valuable model for harnessing the innate immune system in innovative and sustainable ways.

## Figures and Tables

**Table 1:** Clinical studies on the protective effect of the BCG vaccine.

	ClinicalTrials.govID	Disease Entity	Study	Location Countries	Phase	Study Start	References
**ANTIVIRAL EFFECTS**	NCT03213405	RSV	Assess Safety, Tolerability and Immunogenicity of the Live Attenuated hRSV Vaccine rBCG-N-hRSV (EVA-VRS01)	Chile	Phase 1	2017	[127]
	NCT04659941	SARS-CoV-2	Use of BCG Vaccine as a Preventive Measure for COVID-19 in Health Care Workers	Brazil	Phase 2	2020	[128]
	NCT04648800	SARS-CoV-2	Effect of BCG Vaccination on the Incidence and Severity of SARS-CoV-2 Infections Among Healthcare Professionals During the COVID-19 Pandemic	Poland	Phase 3	2020	[129]
	NCT04439045	SARS-CoV-2	Efficacy and Safety of VPM1002 (rBCG) in Reducing SARS-CoV-2 (COVID-19) Infection Rate and Severity	Canada	Phase 3	2020	[130]
	NCT02114255	Influenza	Effects of BCG on Influenza Induced Immune Response	Netherlands	Phase 2 Phase 3	2014	[131]
**CANCER**	NCT06462001	Bladder Cancer	Adding Mitomycin C to BCG in High-risk, Non-muscle-invasive Bladder Cancer	United Kingdom	Phase 3	2020	[132]
	NCT06441110	Bladder Cancer	Effectiveness and Safety of Instillation of BCG afor Intermediate and High-risk Non-muscle Invasive Bladder Cancer	China	Phase 3	2023	[132]
**CANCER**	NCT01838200	Melanoma	Intralesional BCG Followed by Ipilimumab in Advanced Metastatic Melanoma	Australia	Phase 1	2014	[133]
	NCT00671554	Melanoma	Melaxin Cancer Vaccine Plus BCG to Treat Malignant Melanoma	United States	Phase 1 Phase 2	2008	[134]
	NCT00006352	Lung Cancer	Monoclonal Antibody Therapy Plus BCG in Treating Patients With Limited-Stage Small Cell Lung Cancer	Netherlands	Phase 3	1999	[107]
	NCT00037713	Lung Cancer	Survival Patients With Limited Disease (LD) Small Cell Lung Cancer Vaccinated With Adjuvant BEC2 and BCG	Not provided	Phase 3	1998	[135]
**AUTOIMMUNE DISEASES**	NCT00202410	Multiple Sclerosis	Efficacy of Anti-Tubercular Vaccination in Multiple Sclerosis	Italy	Phase2 Phase 3	2001	[136]
	NCT05866536	Type 1 diabetes	Repeat BCG Vaccinations for the Treatment of NewOnset Type 1 Diabetes in Children	United States	Phase 2	2023	[137]
	NCT05180591	Type 1 diabetes	Repeat BCG Vaccinations For The Treatment Of Pediatric Type 1 Diabetes	United States	Phase 2	2022	[138]
	NCT02081326	Type 1 diabetes	Repeat BCG Vaccinations for the Treatment of Established Type 1 Diabetes	United States	Phase 2	2015	[139]

## Data Availability

The data presented in this study are openly available under reference numbers.
